# Pediatric Residency Point-of-Care Ultrasound Training Needs Assessment and Educational Intervention

**DOI:** 10.7759/cureus.28696

**Published:** 2022-09-02

**Authors:** Piyawat Arichai, Marc Delaney, April Slamowitz, Roberto Rosario, Heather Gordish-Dressman, Sonali Basu, Jeremy Kern, Angela Maxwell, Alyssa Abo

**Affiliations:** 1 Pediatrics, Children's National Hospital, Washington, USA; 2 Pediatrics, George Washington University School of Medicine and Health Sciences, Washington, USA; 3 Pediatric Critical Care Medicine, Children's National Hospital, Washington, USA; 4 Hospital Medicine, Children's National Hospital, Washington, USA; 5 Pediatric Emergency Medicine, Children's National Hospital, Washington, USA

**Keywords:** retention, barrier, knowledge, needs assessment, attitude, curriculum, education, pediatric residency, pediatric, point-of-care ultrasound

## Abstract

Background

Prior studies showed that point-of-care ultrasound (POCUS) training is not commonly offered in pediatric residency. We assessed the need for a pediatric POCUS curriculum by evaluating pediatric trainees’ attitudes toward the use of POCUS and identifying barriers to training. We also aimed to evaluate the impact of a POCUS educational intervention on self-efficacy and behavior.

Methods

We conducted a cross-sectional survey of pediatric residents in a single large freestanding children’s hospital distributed via an institutional listserv and administered online. The survey included opinion-rating of statements regarding POCUS and barriers to training. We also offered a two-week POCUS course with online modules and hands-on scanning. Participating residents completed pre- and post-course knowledge assessments and follow-up surveys up to 12 months following the course to assess POCUS use and self-report confidence on POCUS indications, acquisition, interpretation, and clinical application.

Results

Forty-nine respondents were included in the survey representing all three pediatric levels with 16 specialty interest areas. Ninety-six percent of trainees reported that POCUS is an important skill in pediatrics. Ninety-two percent of trainees reported that residency programs should teach residents how to use POCUS. The most important perceived barriers to POCUS training were scheduling availability for POCUS rotations and lack of access to an ultrasound machine. Fourteen participants completed the pre- and post-course knowledge tests, with eight and six participants also completing the six- and 12-month follow-up surveys, respectively. Self-ratings of confidence were significantly improved post-intervention in indications (P = 0.007), image acquisition (P = 0.002), interpretation (P = 0.002), and clinical application (P = 0.004). This confidence improvement was sustained up to 6-12 months (P = 0.004-0.032). Participants also reported higher categorical POCUS use after course completion (P = 0.031).

Conclusions

Pediatric trainees perceive POCUS as an important skill, hold favorable opinions towards the use of POCUS, and support POCUS training within a pediatric residency. A POCUS course can improve resident POCUS knowledge, instill confidence, and motivate higher POCUS use. Further study is needed to evaluate POCUS applications in pediatric medicine to develop a standardized POCUS curriculum and establish a training guideline for pediatric residency.

## Introduction

Point-of-care ultrasound (POCUS) is a focused ultrasound examination performed and interpreted by a provider to interrogate specific clinical questions or to guide procedures. Recent advances in POCUS technology have improved functionality, portability, and affordability, which have enabled wider clinical applications [1-4]. Prior studies show that POCUS-certified providers can perform sonographic studies with similar accuracy compared to sonographers and gold standard modalities [5-8]. As the utility of POCUS in medical care expands, ultrasonography training is being integrated into undergraduate medical education [9-14]. In a similar fashion, POCUS is being adopted more broadly in pediatric disciplines including pediatric emergency medicine (PEM), pediatric anesthesia, pediatric critical care, and hospitalist medicine among others [15-17].

While POCUS education has been developed for pre-clinical settings and subspeciality clinical applications, there is a gap in POCUS training in pediatric residency. A national survey of residency programs found that only 12.4% of pediatric residency programs offer a POCUS curriculum [18]. There is currently no consensus or guidelines on POCUS training for pediatric residents. Thereby, the scope and format of POCUS training among resident programs are variable but are generally isolated educational opportunities without an explicit goal of achieving POCUS competency longitudinally. POCUS competency involves the operator being able to recognize when POCUS is indicated, how to acquire appropriate images, interpret them accurately, and apply the findings to the clinical context. For POCUS competency to be achieved, we need effective educational interventions at the trainee level.

Our primary aim is to assess the need for POCUS training by evaluating the attitude of pediatric residents towards POCUS and barriers to training. Our secondary aim was to inform POCUS curriculum planning by assessing the short-term and long-term effectiveness of a short POCUS educational intervention during pediatric residency.

## Materials and methods

Assessment survey

We performed a cross-sectional survey using a standardized questionnaire. The survey quantitatively assessed the attitude of pediatric residents towards POCUS at a single tertiary care pediatric hospital. The pediatric program had 117 pediatric residents at the time the survey was administered between April 3, 2020 and May 24, 2020.

The survey included demographics, evaluation of attitude assessment, and evaluation of training barriers. The attitude was quantitatively assessed using the 5-point Likert’s rating of agreement (strongly disagree, disagree, neutral, agree, strongly agree) on statements regarding POCUS. POCUS training barriers were assessed using the 5-point Likert’s rating of significance (not at all, slightly, somewhat, very, extremely). The questionnaire was reviewed by residency program faculty and POCUS faculty to optimize face validity. Informed consent was provided, and consent was implied through survey participation. Participation in the survey was voluntary. Identifiable information was not obtained to maintain anonymity. The surveys were administered online and distributed via an email listserv to all pediatric residents at the time of survey administration.

Educational intervention 

We offered a two-week long POCUS course to pediatric residents over two years (October 2018-October 2020). The course consisted of a self-paced online curriculum and hands-on scanning experience. The curriculum used pre-curated content from SonoSim, Inc. The module topics were selected to offer broad POCUS knowledge applicable to pediatric clinical settings. The core clinical topics were: fundamentals of ultrasound, cardiology, pulmonary, musculoskeletal, renal, soft tissue, Extended Focused Assessment with Sonography in Trauma (eFAST), and Rapid Ultrasound in Shock and Hypotension (RUSH). The procedure topics were: introduction to ultrasound-guided procedures and peripheral venous access. Each module involves a two- to three-hour activity including viewing video lessons, completing a knowledge check for each section, and passing a 20-question mastery test. The content of each module includes anatomy, ultrasound imaging technique, clinical application, and literature review. In addition to self-directed learning, residents also completed three to six scanning shifts in the Pediatric Emergency Department with faculty trained in POCUS. During these three-to-four-hour long sessions, residents applied their knowledge to perform POCUS studies on patients to answer specific clinical questions using the SonoSite ultrasound machine. Verbal consent was obtained from each patient and legal guardian to perform the educational ultrasound studies. POCUS faculty reviewed all imaging studies weekly during the Quality Assurance process to check for accuracy.

Effectiveness assessment

The POCUS competencies were defined as the ability to perform the following tasks: 1) identify the clinical indication for the examination, 2) demonstrate the ability to acquire and optimize images, 3) interpret the ultrasound findings accurately, and 4) integrate the findings for clinical decision making [19]. The course participants completed a survey at four separate time points: prior to the course enrollment, at course completion, at six months follow-up, and at 12 months follow-up. The pre- and post-course survey included a POCUS knowledge test consisting of multiple-choice questions aimed to assess participants’ ability to recognize standard POCUS views, identify relevant anatomy, interpret findings, and incorporate findings with the clinical vignette. The questionnaires utilized both still images and video clips of de-identified ultrasound studies. All surveys also included a self-efficacy assessment of confidence in each of the POCUS competencies. We used a continuous variable confidence rating scale from 0% to 100%. Informed consent was provided prior to course enrollment. Participation in the survey was voluntary. Consent was implied through survey participation. The surveys were administered online and distributed via email with personalized survey links.

Data management and analysis

Study data were collected and managed using Research Electronic Data Capture (REDCap) hosted at Children’s National Hospital (CNH). Responses were anonymous without identifiable personal information. No monetary incentives were offered for survey completion. The study was approved by CNH’s Institutional Review Board (IRB). Informed consent was obtained from all study participants.

Data were analyzed using Stata Statistical Software and GraphPad by Prism. The survey on pediatric residents’ attitude toward POCUS was analyzed using descriptive statistics. Categorical data are expressed as numbers and percentages. Continuous data are expressed as mean with standard deviation, median, and range. The impact of the POCUS elective on residents’ confidence and POCUS use was analyzed using mixed effects ANOVA and Chi-square.

## Results

Survey of pediatric residents

The overall response rate was 42% (n=49/117) representing all pediatric training levels: 36.7% PL1, 24.5% PL2, and 38.8% PL3 (Table 1). Resident respondents represented a variety of specialty interest areas: 26% general pediatrics, 22% emergency medicine (EM), 18% neonatal-perinatal medicine, 16% critical care, 12% hospitalist medicine, and 58% pediatric subspecialties. Most of the respondents have had some POCUS exposure: 57% had POCUS training prior to residency, and 57% had POCUS training during residency. Of the participants, 49% have performed POCUS on a patient.

**Table 1:** Descriptive characteristics of survey responders

Characteristic	N (%)
Residency year	
PL1	18 (36.7%)
PL2	12 (24.5%)
PL3	19 (38.8%)
Specialty interest	
Adolescent	1 (2%)
Child abuse	2 (4%)
Critical care	8 (16%)
Developmental-Behavioral	0 (0%)
Emergency medicine	11 (22%)
Endocrinology	0 (0%)
Gastroenterology	1 (2%)
General pediatrics	13 (26%)
Hematology/Oncology	2 (4%)
Hospice and palliative care	0 (0%)
Hospitalist medicine	6 (12%)
Infectious disease	3 (6%)
Neonatal-perinatal medicine	9 (18%)
Nephrology	1 (2%)
Pediatric cardiology	6 (12%)
Pulmonology	2 (4%)
Rheumatology	1 (2%)
Sleep medicine	0 (0%)
Sports medicine	0 (0%)
Toxicology	0 (0%)
Transplant Hepatology	1 (2%)
Other	9 (18%)
Prior POCUS training	
No	21 (43%)
Yes	28 (57%)
POCUS training during residency	
No	21 (43%)
Yes	28 (57%)
Ever performed an ultrasound on a patient	
No	25 (51%)
Yes	24 (49%)
Do you see any barrier to POCUS training	
No	21 (44%)
Yes	27 (56%)

When asked if POCUS should be used for patient care more often, 98% of residents agreed (Table 2). Most (96%) residents agreed that POCUS was an important skill in pediatrics and 92% of residents agreed that pediatric residency programs should teach residents how to use POCUS. Regarding future careers, 88% of residents agreed that POCUS skills will be more important in the practice of medicine in the future, 85% disagreed that POCUS was only important in resource-limited settings, and 88% of residents agreed that POCUS skills will make them stronger candidates for fellowship and job position. Most residents (90%) showed interest in learning POCUS during residency, but a smaller proportion (56%) agreed that POCUS skills should be a core procedure competency for the pediatric residency program.

**Table 2:** Distribution of responses to specific statements using Likert’s five-point scale

Question	Strongly Agree/ Agree	Neutral	Strongly Disagree/ Disagree
POCUS use for patient care			
POCUS should be used for patient care more often	46 (98%)	1 (2%)	0 (0%)
POCUS will help me in the care of my patients	42 (88%)	6 (13%)	0 (0%)
In the past month, I have cared for a patient whose a POCUS study could have been beneficial	35 (73%)	7 (15%)	6 (13%)
I would use POCUS regularly if I had access to an ultrasound machine	34 (71%)	11 (23%)	3 (6%)
I want to use POCUS regularly for patient care	33 (69%)	12 (25%)	3 (6%)
POCUS is only important in resource-limited settings	5 (10%)	2 (4%)	41 (85%)
POCUS in pediatric medicine and future careers			
I think POCUS is an important skill in pediatrics	46 (96%)	2 (4%)	0 (0%)
POCUS skills will be more important in the practice of medicine in the future	42 (88%)	6 (13%)	0 (0%)
POCUS skills will make me a stronger candidate for my fellowship/job position	42 (88%)	4 (8%)	2 (4%)
POCUS training			
Pediatric residency programs should teach residents how to use POCUS	44 (92%)	4 (8%)	0 (0%)
I am interested in learning POCUS during residency	43 (90%)	4 (8%)	1 (2%)
POCUS skills should be a core procedure competency for the pediatric residency program	27 (56%)	14 (29%)	7 (15%)
POCUS training balancing measures			
I would be afraid to miss important findings if I use POCUS	31 (66%)	11 (23%)	5 (11%)
POCUS would replace the need for formal ultrasound studies	6 (13%)	9 (19%)	33 (69%)
POCUS will lead to less need of ionizing radiation studies such as x-ray or CT scans	36 (75%)	9 (19%)	3 (6%)
Current state of POCUS training			
There is plenty of opportunity to learn POCUS at Children's National	13 (27%)	17 (35%)	18 (38%)
It is easy to find an ultrasound machine at Children's National	4 (8%)	10 (21%)	34 (71%)

The POCUS studies that were most rated as being useful in pediatric patients were bladder volume assessment, presence of pericardial effusion, presence of pleural effusion, and vascular access guidance (Table 3). Of the respondents, 56% reported seeing at least one barrier to POCUS training. Factors that are rated from most to least significant included: 1) scheduling availability for POCUS rotation, 2) lack of access to ultrasound machines, 3) time needed to learn, 4) availability of instructors, 5) lack of IT infrastructure and 6) lack of interest in faculty to train residents (Table 4).

**Table 3:** Proportion of respondents who perceive the following POCUS applications as useful

POCUS application	N (%)
Qualitative assessment of bladder volume	48 (98%)
Presence and degree of pericardial effusion	46 (94%)
Presence of pleural effusion	45 (92%)
Central venous catheter insertion	43 (88%)
Assessment of the testicles	41 (84%)
Peripheral IV insertion	40 (82%)
Arterial line insertion	40 (82%)
Presence of lung consolidation	39 (80%)
Presence of musculoskeletal abscess	39 (80%)
Assessment of the appendix	38 (78%)
Qualitative assessment of joint effusion	38 (78%)
Presence of lower extremity thrombus	36 (73%)
Presence of fluid in the peritoneum	36 (73%)
Lumber puncture guidance	36 (73%)
Assessment of the gall bladder	34 (69%)
Presence of fluid in the pelvis	34 (69%)
Assessment of the ovaries	34 (69%)
Presence of intussusception	33 (67%)
Presence of pyloric stenosis	32 (65%)
Presence of pneumothorax	31 (63%)
Qualitative assessment of the global systolic function	30 (61%)
Qualitative assessment of the hydronephrosis	29 (59%)
Presence of pulmonary edema	26 (53%)
Presence of lymphadenopathy	26 (53%)
Qualitative assessment of the IVC as indicator of hypovolemia	24 (49%)
Assessment of proper IUD placement	22 (45%)

**Table 4:** Distribution of the level of significance of the following barriers to POCUS training using Likert’s five-point scale

Factor	Response N (%)		
	Not at all/Slightly	Somewhat	Very/Extremely
Scheduling availability for POCUS rotation	3 (9%)	10 (32%)	18 (58%)
Lack of access to machines	4 (13%)	9 (29%)	18 (58%)
Time needed to learn	5 (16%)	10 (32%)	16 (52%)
Availability of instructors	8 (25%)	11 (35%)	12 (39%)
Lack of IT infrastructure	8 (26%)	11 (35%)	12 (39%)
Lack of interest to train	17 (57%)	5 (17%)	8 (26%)

Educational intervention

**Figure 1:**
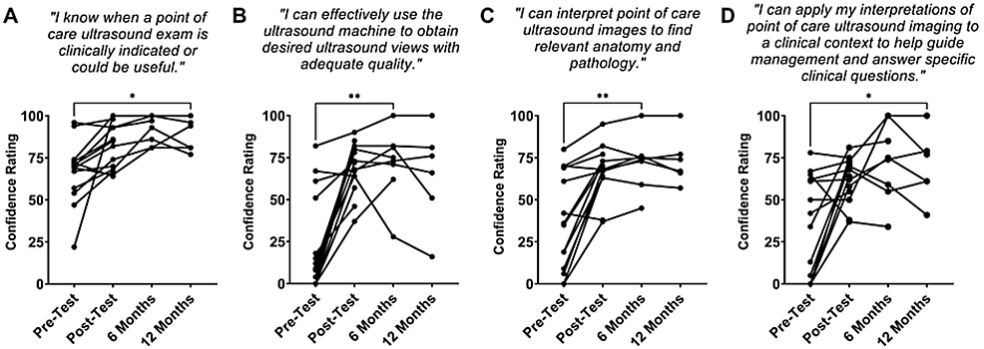
Brief POCUS education at the resident level instills longitudinal confidence in key POCUS educational objectives. Self-ratings on a scale of 0 (no confidence) to 100 (full confidence) in key realms of POCUS indications (A) as well as image acquisition (B), interpretation (C), and application to clinical context (D), with survey questions abstracted above. Each key area demonstrated statistically significant improvement with course completion (Mixed effect ANOVA, P < 0.01 for A-D). The latest follow-up interval of statistically significant improvement from the pre-test is denoted above the graph (P < 0.05). POCUS - Point-of-Care Ultrasound

**Figure 2:**
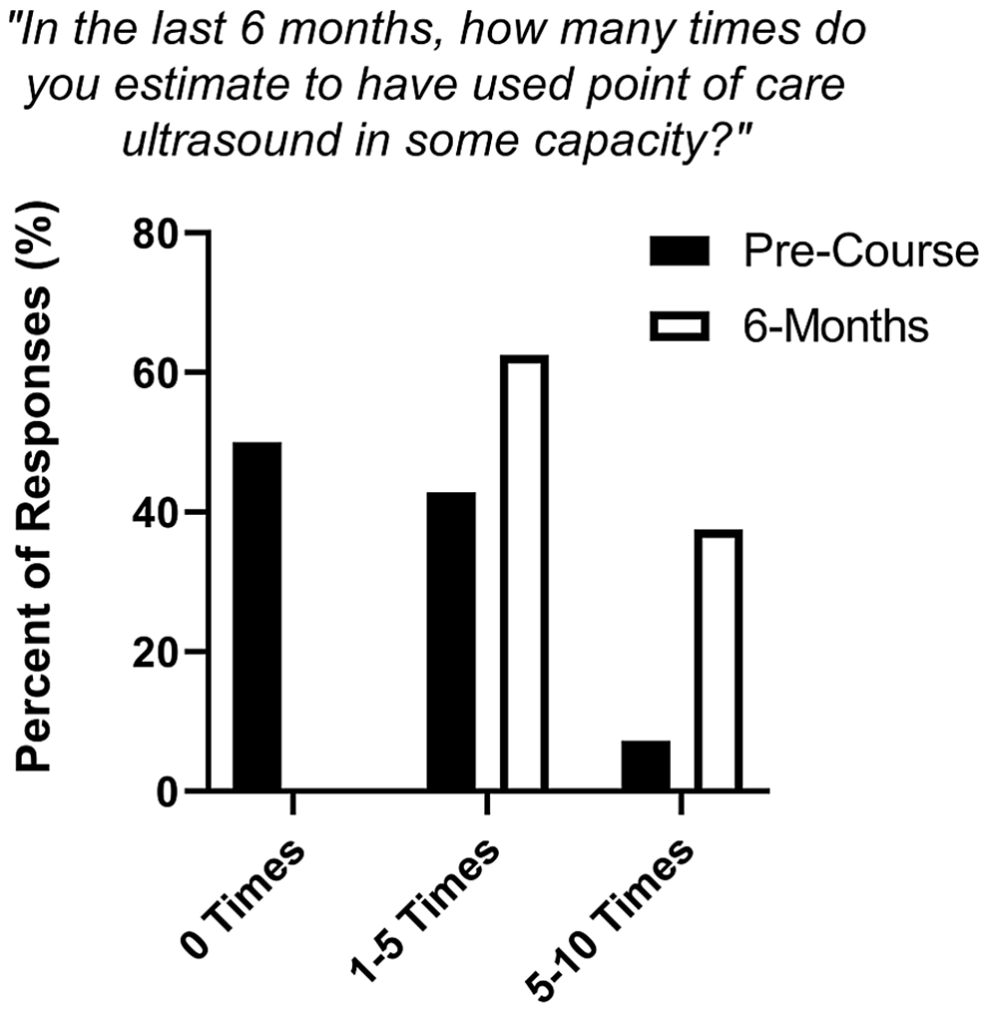
Resident POCUS course graduates self-report higher POCUS use after course completion. Categorical self-rating of POCUS use represented as percent of responses from pre-test (n=14) to six-month follow-up survey (n = 8) (Chi-square, p = 0.03), with survey question wording abstracted above chart. POCUS - Point-of-Care Ultrasound

Residents who participated in the two-week focused POCUS rotation demonstrated improvement in all four POCUS competencies: 1) POCUS indication, 2) image acquisition, 3) interpretation and 4) clinical application (Mixed effect ANOVA, P < 0.01). Residents reported a sustained improved level of confidence in these four areas from pre-test level at six-month and 12-month follow-up (P < 0.05) (Figures 1A-1D). At six months follow-up, residents reported higher level of POCUS use from pre-test (Chi-square, p = 0.03; Figure 2).

## Discussion

Overall, our study demonstrated a strong need for POCUS training in pediatric residency and a two-week POCUS course was effective at broadly raising residents’ confidence in POCUS skills. Pediatric trainees are interested in obtaining POCUS training during residency and view POCUS as an important skill for their future careers. Our finding is consistent with other needs assessment studies indicating that pediatric residents increasingly demonstrate an interest in POCUS [18,20-23]. Upstream of residency training, medical schools are also incorporating ultrasound training into undergraduate medical education [14,24-31]. As this trend continues, future pediatric residents will likely have more familiarity with POCUS and likely seek training for its clinical applications. Therefore, we anticipate that the demand for POCUS training in pediatric residency is likely to increase further in the future. 

The two-week POCUS course demonstrated a significant improvement in self-reported POCUS knowledge and skill among participants, with lasting effects during the six-month and 12-month follow-ups. Participants also reported a higher frequency of POCUS use post-elective compared to prior to the elective. These results show that a brief educational intervention consisting of asynchronous learning and hands-on scanning time with POCUS-trained faculty are effective in improving knowledge and skill in the short term and suggest that the effect may last up to 12 months. 

Our curriculum was easy to develop given the use of pre-existing online POCUS education and the existing POCUS scan shifts in the emergency department (ED) with POCUS-trained PEM faculty. Only one to two residents were offered the elective in each block to maximize the hands-on scanning experience. Given this structure, the asynchronous learning platform was helpful to provide residents with the background POCUS education while allowing faculty to focus on coaching at the bedside. This brief POCUS curriculum was designed to be an introduction to the clinical applications of POCUS in pediatrics rather than to create POCUS competency. To create a level of competency necessary for trainees to be able to use POCUS for patient care, a longitudinal POCUS curriculum will be required [22]. 

Our needs assessment identified barriers in POCUS training that pediatric residency programs need to consider. Firstly, the physical infrastructure, including ultrasound equipment and storage system for image archive and quality assurance process, must be obtained. This was a major barrier in our program, as residents only had access to an ultrasound device in the ED and pediatric intensive care unit (PICU). These ultrasound devices also need to be connected to an image storage system for studies to be reviewed by POCUS-trained faculty for quality assurance. 

Another major barrier is the limited number of POCUS-trained physicians. The experience of one longitudinal POCUS curriculum shows that few residents obtain POCUS studies outside the training sessions because they feel uncomfortable using POCUS for patient care without a supervisor [23]. Without opportunities to perform POCUS scans in the clinical environment, trainees will likely lose knowledge and skill over time. One way to address this shortcoming is to increase training and credentialing programs for pediatric attendings [32,33].

Limitations of our needs assessment included: the use of single-institution data, potential sampling bias, limited response rate, and survey fatigue. Due to the convenience sampling method, our study sample may not represent the view of the pediatric residents in the United States and therefore may not be generalizable to other pediatric training programs. We anticipate that trainees who are interested in POCUS are more likely to voluntarily participate in the study leading to a positive bias. However, our findings are consistent with other prior needs assessments indicating a strong interest in POCUS training among pediatric trainees [21].

The evaluation of our educational intervention is self-efficacy data, which is a lower-level learning goal. Alternatives would be to evaluate individuals’ image acquisition and interpretation skills through standardized assessment or measuring the impact on patient care outcomes, which would be higher-level learning goals. Additionally, the data may be limited by the relatively small sample size and potential selection bias of pediatric trainees who are more interested in POCUS. However, our data included a large proportion of trainees without prior POCUS training. The small sample size of our POCUS course participants is due in part to the logistical constraints of the elective availability. The elective focuses on POCUS scanning on actual patients in real clinical scenarios, thereby the elective can only be offered to one to two residents each two-week block to allow for maximum hands-on experience. 

The larger implication of our study is to assess the need for guidelines on a core set of POCUS competencies for pediatric residency training. In EM, POCUS is a core competency of the residency training that is mandated by the Accreditation Council for Graduate Medical Education (ACGME) [34]. A multiorganizational committee of representatives established the core skills, competency assessment, and education structure for POCUS training in EM residency [35]. In pediatrics, the American Academy of Pediatrics (AAP) provided a guideline for establishing a formal POCUS program for pediatric emergency [36]. Recent articles provided a summary of evidence and specific applications of POCUS in PEM, pediatric hospitalists, pediatric intensive care, and neonatal intensive care [17,36-41]. As pediatric residency training is shaped by the practice of the core pediatric disciplines, the general pediatric residency POCUS curriculum needs to be guided by the POCUS competencies of all the disciplines where trainees spend most of their time in training. However, there is currently no pediatric discipline other than PEM that has established a census on the core set of POCUS competencies for fellowship training [42]. Furthermore, each discipline and program need to develop its own regulation for establishing training competency and credentialing processes, which is a resource-intensive process. Pediatric residency POCUS training programs require navigating the uneven infrastructure among pediatric specialties and developing a competency assessment process that is both unique to general pediatric training and in harmony with the existing institutional processes of the various internal departments.

## Conclusions

Pediatric trainees perceive POCUS as an important skill, hold favorable opinions toward the use of POCUS, and support POCUS training within the pediatric residency. A two-week POCUS course with asynchronous learning and hands-on scanning instills confidence and POCUS use. Further study is needed to evaluate POCUS applications in general pediatric medicine to develop a standardized POCUS curriculum and establish a training guideline for pediatric residency.
